# Supervised Classification of Operator Functional State Based on Physiological Data: Application to Drones Swarm Piloting

**DOI:** 10.3389/fpsyg.2021.770000

**Published:** 2022-01-06

**Authors:** Alexandre Kostenko, Philippe Rauffet, Gilles Coppin

**Affiliations:** ^1^UMR 6285 Laboratoire des Sciences et Techniques de l’Information, de la Communication et de la Connaissance (LAB-STICC), Brest, France; ^2^FHOOX Team, Lab-STICC, Université Bretagne Sud, Lorient, France; ^3^CROSSING IRL CNRS 2010, Adelaide, SA, Australia

**Keywords:** Physiological data, mental state classification, drone operation, machine learning, mental workload

## Abstract

To improve the safety and the performance of operators involved in risky and demanding missions (like drone operators), human-machine cooperation should be dynamically adapted, in terms of dialogue or function allocation. To support this reconfigurable cooperation, a crucial point is to assess online the operator’s ability to keep performing the mission. The article explores the concept of Operator Functional State (OFS), then it proposes to operationalize this concept (combining context and physiological indicators) on the specific activity of drone swarm monitoring, carried out by 22 participants on simulator SUSIE. With the aid of supervised learning methods (Support Vector Machine, k-Nearest Neighbors, and Random Forest), physiological and contextual are classified into three classes, corresponding to different levels of OFS. This classification would help for adapting the countermeasures to the situation faced by operators.

## Introduction

Many operators carry out their activity in complex, high-risk situations and with strong time pressure. This is particularly the case in air domain, for fighter pilots (Veltman and Gaillard, 1996; Lassalle et al., 2017) or for drone operators (Pomranky and Wojciechowski, 2007; Kostenko et al., 2016). More specifically concerning the drone operations, piloting drones currently requires one or more operators for a single drone: for example, Cummings et al. (2007) recall that the predator and the shadow require two operators. Nevertheless, in the next generation of UAV systems, a ground operator will be required to supervise several UAVs cooperating to achieve their mission (Johnson, 2003; Coppin and Legras, 2012). According to Wickens et al. (2005), the management of several drones can cause serious problems of mental workload or attentional tunneling, that can ultimately lead to errors. Improving safety and performance of risky missions carried out by these operators becomes therefore an important challenge. This one could be solved by adjusting in real time the dialogue and the cooperation between man and machine according to the state of the human operator (Dixon et al., 2005; Wickens et al., 2005; Kostenko et al., 2016). It therefore becomes crucial to assess online the operator’s ability to keep performing the mission, to anticipate potential performance impairments, as well as to activate appropriate countermeasures in time (change in system transparency level, dynamic function allocation, etc.). In this context, a collaboration project was conducted with Dassault Aviation.

To encapsulate the different elements contributing to a potential degradation of performance from an operator, Hockey (2003) proposes the notion of OFS, namely Operator Functional State. This concept is defined as “the variable capacity of the operator for effective task performance in response to task and environmental demands, and under the constraints imposed by cognitive and physiological processes that control and energize behavior.” This definition first underlines a strong relationship between the OFS and his/her performance on the tasks, leading to OFS classification using categories like “Capable/Incapable,” “Low risk/Very risky,” or more generally classes expressing a gap to expected performance. However, it is often difficult to predict a performance collapse of an operator solely based on the analysis of the results of his/her activity. This difficulty is particularly pregnant for experienced operators: the observable degradations of their performance are indeed only slight and gradual (before a stall) since these operators have regulatory strategies to maintain during a certain time the effectiveness of the main tasks. Therefore, the time of reaction and adaptation of the system to a performance collapse may be too long to be caught for the situation and may thus cause irreversible effects (Yang and Zhang, 2013). Thus, the OFS concept aims at coping with these difficulties for anticipating a decrease in operator performative capacity.

Recent works related to the classification of OFS [(Hancock et al., 1995; Noel et al., 2005; Bergasa et al., 2006; D’Orazio et al., 2007; Hu and Zheng, 2009; Kurt et al., 2009; Liu et al., 2010; Zhang and Zhang, 2010; Khushaba et al., 2011; Sahayadhas et al., 2012; Wang et al., 2012; Zhao et al., 2012; Bauer and Gharabaghi, 2015; Kavitha and Christopher, 2015, 2016; Zhang et al., 2015; Gagnon et al., 2016; Gilani, 2016), see Table 1 in section “Methods for Operator Functional State Classification” for comparative review] has shown that supervised learning can be effectively used to detect different levels of OFSs from physiological indicators, using different classifiers (like Support Vector Machine, k-NN, Neural Network). These first results, however, were based on a very discrete classification, that often provides a binary categorization (functional or non-functional operator state). Moreover, all these research works used an *a priori* task difficulty level to supervise the learning of physiological data, often without checking the validity of this task difficulty level regarding the subjective experience of the participants, or without considering the finer-grained variations of task difficulty within complex and dynamic situations.

**TABLE 1:** Methods applied to OFS classification (Hancock et al., 1995; Noel et al., 2005; Bergasa et al., 2006; D’Orazio et al., 2007; Hu and Zheng, 2009; Kurt et al., 2009; Liu et al., 2010; Zhang and Zhang, 2010; Khushaba et al., 2011; Sahayadhas et al., 2012; Wang et al., 2012; Zhao et al., 2012; Bauer and Gharabaghi, 2015; Kavitha and Christopher, 2015, 2016; Zhang et al., 2015; Gagnon et al., 2016; Gilani, 2016).

Authors	Data	Number of classes	Classification method	Accuracy
Wilson and Russell (2003)	EEG, DP, HR, BR	3	NN	Individually: 84%
Zhang et al. (2015)	Gaze position, PD, performance	2	Decision tree	Individually: 81%
Noel et al. (2005)	HRV, HR, Blinkf and EEG	3	Neural network	Individually: 80%
Bergasa et al. (2006)	PERCLOS, BLKf, BLKd, gaze fixations	3 or 5	Fuzzy classifier	Individually: 80% (BLKf)–95% (fixations)
D’Orazio et al. (2007)	BLKd and BLKf	3	kNN	Individually: 95%
Kurt et al. (2009)	EEG, EoG, EMG	3	NN	All participants: 97–98%
Hu and Zheng (2009)	EoG, EMG	3	SVM	All participants: 90%
Zhang and Zhang (2010)	PERCLOS		SVM (Support Vector Machine)	All participants: 99%
Liu et al. (2010)	EEG	2	Hidden Markov Model	All participants: 84%
Khushaba et al. (2011)	EEG, ECG, EoG	2	LDA, LIBLINEAR, kNN, SVM	All participants: 95–97%
Yin and Zhang (2014)	EEG, ECG, and EOG	2	LS-SVM	Individually: 93%
		3	SVM (1 vs. 1)	Individually: 72%
Wang et al. (2012)	EEG	3	Neural network	Individually: 80% All participants: 58%
			NB (Naive Bayes)	Individually: 79% All participants: 43%
Zhang et al. (2015)	EEG, ECG, and EOG	3	SVM	Individually:69%
		4	SVM	Individually: 56%
Kavitha and Christopher (2016)	HRV	5	SVM	All participants: 95%
Gilani (2016)	HRV, EEG	2	kNN	All participants: 81%
			SVM	All participants: 56%
			RF (Randon Forest)	All participants: 87%
Gagnon et al. (2016)	HRV, BR, gaze position, PD, BLKf	2	kNN	Individually: 91% All participants: 89%
			Neural network	Individually: 83% All participants: 84%
			SVM	Individually: 84% All participants: 83%
			Decision tree	Individually: 85% All participants: 83%

*EEG, Electro Encephalo Graphy; BR, Breathing Rate; ECG, Electro Cardio Gram; HR, Heart Rate; HRV, Heart Rate Variability; PD, Pupil Diameter; EoG, Electro-oculaGraphy; PERCLOS, PERcentage of Eyelid Closure; BLKd/BLKf, Blink duration and frequency; EMG, ElectroMyoGraphy.*

This article aims therefore at developing a more robust method for classifying physiological data in three different classes, corresponding to three different OFS level. This classification is based on the learning of physiological data that can easily be implemented online and in field conditions: cardiac signals and eye metrics. Moreover, to supervise the data learning, we aim at providing an objective and dynamic task difficulty indicator.

This article starts by a literature review that allows to present and choose the most relevant physiological data and classification methods for producing a supervised learning of OFS based on physiological indicators. Then the section “Materials and Methods” deals with two aspects: (i) experimental data acquisition is presented, by describing the use case carried out on a drone swarm simulator, and the dataset that will be used as input for OFS classification; (ii) the supervised classification approach is also described, by explaining how a task difficulty indicator was defined to label physiological data, and how the chosen tested classification methods were parameterized and trained. This approach is implemented in the section “Results,” then finally discussed, by especially pointing out the potential use of this OFS classification to go toward adaptative interfaces and automations, sensitive to what an operator is experiencing during his/her activity.

## Literature Review

### Mental Workload to Approach Operator Functional State

According to Hockey (2003), OFS can be attached to underlying cognitive states, and it is especially associated to mental workload (Yin and Zhang, 2014; Parent et al., 2016). Mental workload is a broad concept that reflects the coherence between the task constraints and the operator’s capacity specific to each individual (Hancock et al., 1995; De Waard, 1996; Kostenko et al., 2016). Following the ergonomics principles of standard DIN ISO 10075-1:2017 (DIN EN ISO 10075-1, 2018), mental workload is viewed from both aspects of mental stress (i.e., the constraints imposed upon operators) and mental strain (i.e., the cognitive cost of the task for the operators). There can therefore be underload if the capacity is not exploited (with a decrease of engagement of the operator), or overload (if the mental strain required to perform the task exceeds the capacities of the operator at a given time). In these both extreme cases, the operator is considered as not functional. On the contrary, when the operator can be effective at a reasonable cognitive cost, the mental workload is acceptable, and the operator is considered as functional. Finally, as suggested by De Waard (1996) and Hockey (2003), some transitional states may exist between the functional state and the non-functional states (e.g., underload/overload). This is the case when an operator succeeds in maintaining task performance, but at a very high cognitive cost reaching his capacity limits. This state is generally transient, leading to mental fatigue, and finally resulting in a decrease of operator capacity and therefore in a potential overload.

### Physiological and Contextual Indicators to Assess Operator Functional State

Operator Functional State can be evaluated by the measurement and the observations of several type of variables: we can especially distinguish neuro-physiological indicators, related to operator’s mental strain, and contextual indicators related to the task constraints (also called mental stress).

#### Neuro-Physiological Indicators of Mental Strain

Since Kahneman’s (1973) energetic approach emphasizing the relationship between physiological activation and mental activity, OFS has been associated with variations in physiological signals [heart rate variability (HRV), electrodermal activity, pupillary diameter, etc.]. Moreover, with Parasuraman (2003) and the rise of neuro-ergonomics, new approaches are focusing on the link between the cognitive states of the operator and the waves generated by brain activity [with devices such as EEG or fNIRS (Strait and Scheutz, 2014)]. Neurophysiological signals are generally not used in raw form, but they initially produce indicators that are then processed by classification algorithms. These indicators are either directly derived from the measurement (as for example the heart rate established by simple observation of the QRS complex) or built after a projection of the measurements in a dedicated representation space (wavelets, etc.). It may also be necessary to incorporate a data cleansing step (e.g., removing RED-NS parasites for electrodermal activity) and normalization of the data against a baseline (Lassalle et al., 2017).

The signals relating to central nervous system (EEG) are very sensitive and very discriminating on the different operating states (Aghajani et al., 2017), but the sensors are for the moment very invasive, and the treatments are relatively complex (aggregation by fusion of data of the different brain regions in temporal and frequency domains), which can limit the real-time application of these techniques. Many recent studies use an EEG or even only work on this sensor, which produce many distinct variables (not necessarily uncorrelated). The only optimization or regression objectives, necessary for the adjustment of parametric class models to supervised machine learning, must thus implement recent methods of structured regression or “pruning” (Tachikawa et al., 2018). The choice of the algorithm and of its parameters to be able to manage noisy and high-complexity signals are still open research questions.

The physiological signals, dependent on autonomic nervous system (HRV, electrodermal activity, and pupillary diameter), make it possible to approach the sympathetic (activation) and parasympathetic (awakening at rest) tendencies of the organism. They are therefore sensitive to mental strain. On the other hand, they have a weak diagnosticity: it is indeed difficult to discriminate using only physiological signals from cognitive states of the same tendency (for example fatigue, attentional focus, or mental workload). It should also be noted that the electrodermal activity is not sensitive enough for short-term treatment: the signal tends to react quickly to a stimulus but decreases slowly when the stimulus disappears (Rauffet et al., 2015). The latency effect is therefore too important to monitor the OFS in real time.

#### Contextual Indicators of Mental Stress

In addition, the weak diagnosticity of some of these data tends to show the need to contextualize the physiological indicators to better determine and categorize the OFS. To face this challenge, several research works propose to “situate” the cognitive states according to the mental stress, i.e., to characterize the constraints of the situation (Durkee et al., 2015; Schulte et al., 2015). These contextual indicators related to task difficulty can be expressed in terms of stimuli density or spatial information disparity at a given time, or in terms of temporal pressure (remaining margins within a time budget).

### Methods for Operator Functional State Classification

To classify OFS from physiological contextual data, different methods were proposed. Kotsiantis et al. (2007) summarizes the main methods of supervised machine learning. Some can only deal effectively with one type of data: discrete data for “Naïve Bayes Classifiers” and “Rule-Learners,” and continuous data for neural networks (NN), k-Nearest Neighbors (kNN), and Support Vector Machines (SVM). Only decision trees can handle both types of data. Moreover, there are relatively recent references to the classification of physiological signals, which allow a slightly more specific analysis (cf. Table 1) applied to OFS classification. In the present study, we seek to classify continuous data (like HRV of pupillary diameter). As “Naïve Bayes Classifiers” and “Rule-Learners” do not effectively process continuous data, they can be discarded from our study. Kotsiantis et al. (2007) also shows that SVM and NN have similar operational profiles (Kotsiantis et al., 2007). These two methods are efficient for processing continuous data and are effective for managing multicollinearity. Both methods require having a large learning database to be effective, but SVMs are more tolerant of missing data, better manage over-learning, and generally have better reliability. Moreover, the synthesis on OFS classification (cf. Table 1) underlines that SVM is the most used algorithm. So, we choose to discard the NN for the benefit of the SVM. In addition, we seek to achieve a multiclass classification (more than two classes). kNN and Random Forest method have the advantage of being of a multi-class nature. It is therefore relevant to implement these two methods. These three methods will be kept and tested, as there is currently no methodological guide to optimally choose a method and its settings.

It should be also noted that most of the works presented in Table 1 only proposed a binary OFS classification. The few works investigating a multiclass classification were based on a single signal [Bergasa et al. (2006) applied a fuzzy classifier to different types of signals taken separately, whereas Wang et al. (2012) and Kavitha and Christopher (2016) obtained effective results with SVM for a 3- or a 5-class classification, but only with one source, HRV or EEG]. In this work, we aim to propose a multiclass classification based on the consideration of several signals, to improve the selectivity and the sensitivity of OFS classifier.

Finally, all these references use an *a priori* evaluation of task difficulty to label the physiological data, based on the manipulation of task parameters (such as the frequency of stimuli presented to participants, or the number of concurrent tasks to carry out at the same time). This approach is questionable and can raise some problem of selectivity, since we are not sure that the task difficulty designed by experimenter really generates significant OFS variations in participants. Therefore, we will look for proposing a dynamic task difficulty indicator subjectively validated by the participants.

## Materials and Methods

This section explains how data was collected, as input for our proposed classifiers (section “Data Acquisition: Application to Drone Swarm Piloting”). Then our classification approach is further developed, by presenting the way we design our task difficulty indicator to label physiological data, and how we train the models by tuning the parameters (section “Classification Approach”).

### Data Acquisition: Application to Drone Swarm Piloting

The acquisition of data, necessary for OFS classification, was carried out on a drone simulator, named SUSIE (Coppin and Legras, 2012). It consists in securing an area by piloting a swarm of drones. The aim of the simulation is to find, identify and neutralize different mobile targets, hidden at the beginning of the simulation. This simulator was chosen because it presents several advantages for our study: it is adapted to the emergent multitasking activity of and operator managing several drones; it is ecological and complex enough to consider the future implementation of proposed OFS classification on real system; and it constitutes a microworld on which the experimenter can control different simulation parameters (task difficulty, target location, and appearance time) and can record behavioral (actions achieved by participants with mouse clicks) and performance indicators (reaction times or number of processed targets over time).

#### Participants

A total of 22 participants, aged from 22 to 30 (mean: 23, standard deviation: 2, 4) took part in the experiment. For reasons of homogeneity, all were men from the same scientific diploma, and had good experience with video gaming (this point was controlled by a short questionnaire). Since there is no expert operator for the monitoring activity of swarm drones (the system is indeed experimental), we considered that this population, with these scientific and practical skills, could represent the future drone operators.

#### SUSIE Simulator

**FIGURE 1:**
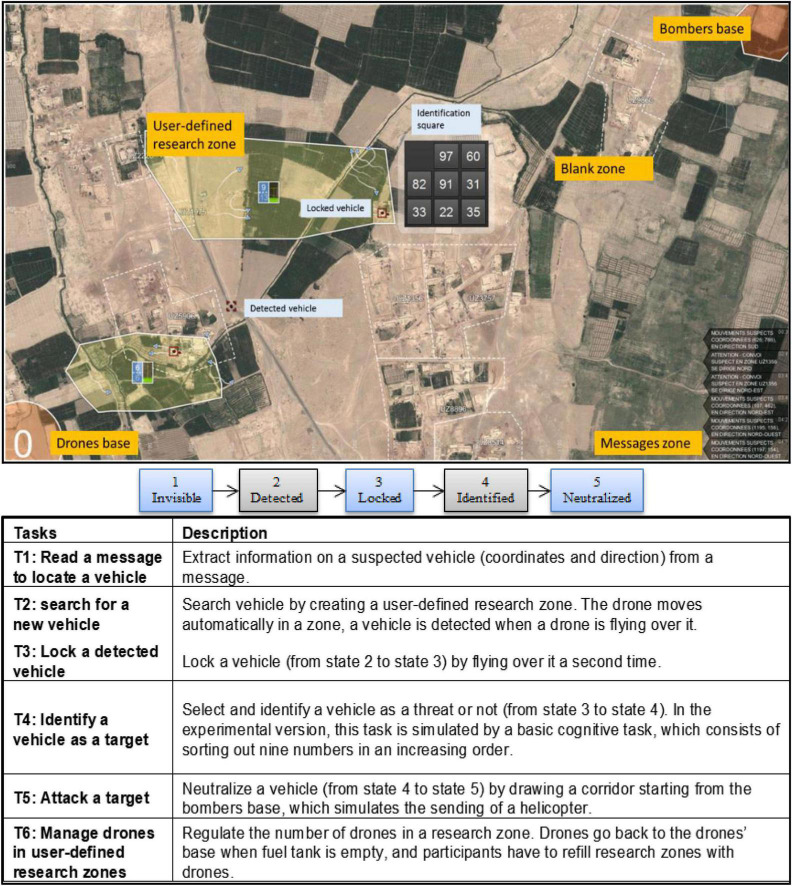
SUSIE environment, sequential target states, and related tasks.

This is a Java based software that allows participants to interact with and to supervise a swarm of drones using a mouse-screen system. Only one operator is required, but some tasks can be or are achieved by an artificial agent. The system provides different information to the operator from two sources: a dynamic map and a message banner on the right (Figure 1, top image). The dynamic map gives information about the areas that the drones control such as the vehicles in these areas and their state. The message banner indicates the coordinates and direction of a vehicle that the operators need to assign high priority to its neutralization. The main task is to detect and neutralize the threats (i.e., hostile vehicles) on the map. When a vehicle is generated by the software, it is hidden, i.e., it is present on the map but invisible (it has to be detected by drones sent by the participant). Before it is neutralized, the status of the vehicle changes several times (Figure 1, middle image). To advance from one status to the next, operators need to complete many sub-tasks, summarized in Figure 1.

#### Scenario and Training

A scenario was designed on the SUSIE prototype to generate variations in OFS. The chosen scenario lasts 25 min and is divided into four phases: a waiting period P1 (2 min, no stimuli), followed by three attack phases with increasing difficulty, respectively P2 (7 min, low difficulty), P3 (7 min of medium difficulty), and P4 (9 min of hard difficulty). The difficulty of the task was modified by varying the frequency of appearance of vehicles and messages on the SUSIE simulator. A 30-min phase preceded the run of this scenario, to calibrate the biofeedback sensors and train the participants. The calibration consists especially in recording a baseline for eye activity and heart rate metrics, by leaving participants during 5 min in front of a white screen without anything to do. The training is divided into two parts: a presentation part (10 min) and a practical trial part (20 min). The presentation consists of explaining the system, giving the objectives and the prescribed operating modes to achieve the objectives. The practical trial consists in allowing participants to take control of the system. During this second part, the recommended procedures were regularly reminded.

#### Equipment

Participants performed the experiment in a room where brightness is controlled and constant, to avoid pupillary reflex due to light variations. In this room a space has been created for running the scenario on the simulator, with fixed desk and chair (to avoid parasitical movements of participants). The system supporting SUSIE software is composed of a 24″ screen and a mouse connected to a PC. Data recordings was done with a SeeingMachine FaceLAB5^®^ eye tracker for pupillary response, a Zephyr Bioharness^®^ heart rate belt, and a log (text file) of scenario events and operator’s mouse actions recorded on SUSIE. Subjective experience of participants was also collected by using questionnaires, popping up every 90 s on screen.

#### Data Selection and Processing

The physiological data (Heart Rate Variability, HRV and Pupillary Diameter PD) were collected with the aid of the equipment described above. HRV was computed from the standard deviation of the NN interval on the last 300 heartbeats (SDNN method), following the formula $s\,q\,r\,t\,\left(\frac{\sum_{i=1}^{N}{\left(R\,R\,i-\text{mRR}\right)}^{2}}{N-1}\right)$. Pupil diameter was cleansed (diameter smaller than 2 mm and larger than 8 mm were excluded). All the physiological data were *z*-normalized to remove the interindividual differences. Moreover, as explained in section “Physiological and Contextual Indicators to Assess Operator Functional State,” these physiological data must be combined with contextual data. To have an accurate and dynamic indicator of task difficulty improving the OFS, we considered the following variables to characterize the dynamic task difficulty within SUSIE simulator: “N1” as the number of targets visible on the screen that must be processed, “N2” as the number of messages visible on the screen that must be processed, and “Entropy” as the spatial entropy of the distribution of targets displayed on the screen (by dividing the screen in 8 equal zones, and calculating ΣPi*Log(Pi), with Pi the proportion of the targets included in each zones on the total number of targets displayed on the screen). Finally, subjective scores were also considered as variables to validate the dynamic task difficulty and the different classes of OFS classification. We use a Likert scale for the level of task difficulty, and an Instantaneous Self-Assessment (ISA) (Tattersall and Ford, 1996) for the OFS.

### Classification Approach

**FIGURE 2:**
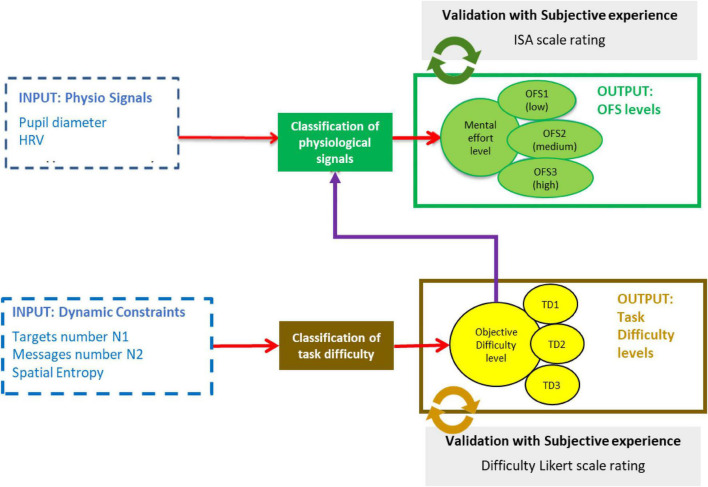
Supervised learning of physiological data.

We first define and validate a dynamic task difficulty indicator, to then supervise the classification of physiological data. Figure 2 summarizes the principle of this classification process that was implemented. We used MATLAB software (Paluszek and Thomas, 2016) and the machine learning libraries (*fitcsvm* and *fitcecoc* for SVM, *treebagger* for Random Forest, and *Classification KNN* for k-NN) to train and test the different machine learning methods.

#### Definition and Validation of a Task Difficulty Indicator to Label Physiological Data

The goal of this first step was to classify a level of task difficulty (TD), based on indicators of task constraint and performance identified in the previous section. This “contextual” indicator will subsequently be used as a label for supervising the learning of physiological states. The computation of the difficulty indicator TD is divided into two stages: first, raw data were processed by discretization into two or three categories: the threshold between these categories were set according to a preliminary study of the subjective experience of participants on different conditions of task difficulty. Moreover, the different discretized variables were initialized at the beginning of the scenario, by considering the constraint variables (N1d, N2d, Entropied, cf. Table 2); then, a data fusion of the discretized indicators was achieved by aggregation, to obtain a global indicator of task difficulty with three categories (TD1 = low, TD2 = medium, TD3 = high). Aggregation rules were defined as follows: if {N1d = High and N2d ≥ Medium and Entropy ≥ Medium} then TD = High.

**TABLE 2:** Data processing for task difficulty indicator.

Variables related to task difficulty	Raw data	Definition	State space	Discretized data	State space
Constraint variables	N1	Represents the number of targets that must be processed	{0, 1,…}	N1d (targets)	low: N1 ≤ 5 medium: 5 <N1 ≤ 11 high: N1> 11
	N2	Represents the number of messages that must be processed	{0, 1,…}	N2d (messages)	low : N2 ≤ 2 high: N2> 2
	Entropy	Represents the spatial entropy of the different targets on the whole monitored space	Continue	Entropyd	low: Entro ≤ 0.45 medium: 0.45 <Entro ≤ 1 high: Entro> 1

To validate the difficulty indicator (TD), we studied the correlation between this indicator and the subjective assessment of the task difficulty, collected every 90 s with a Likert scale. As the difficulty indicator TD and the subjective assessment are ordinal qualitative variables, a Spearman test was therefore used to study the correlation. It showed that these two variables are positively correlated (*r* = 0.711, *p* < 0.001). The task difficulty indicator TD can therefore be used to supervise the classification of physiological signals.

#### Supervised Classification Method

The aim of this stage is to classify the OFS into three categories, by applying a supervised learning of the physiological signals with the three different task levels defined in Stage 1.

Two main physiological indicators, pupillary diameter and HRV, were selected for this classification of OFS, based on the literature review of section “Physiological and Contextual Indicators to Assess Operator Functional State.”

The accuracy of the three supervised learning methods identified in the literature (SVM, kNN, and Random Forest) are evaluated by using an 75/25% train-test split, and by applying a fourfold cross-validation procedure.

For each of these methods, we tested different settings (cf. Table 4 for the best settings).

For SVM, we used several kernel methods, under different parameters of *gamma* and *coeff.*

• Polynomial kernel: K(u,v) = tanh(*gamma* * u′v + *coeff*),
• RBF kernel: K(u,v) = exp (−*gamma* * | | u-v| | ^2^),
• Sigmoid kernel: K(u,v) = (*gamma* * u′v + *coeff*)^d^.

We also tested several types of distance measurements (Euclidean, Euclidean squared, Manhattan, and Chebyshev) and several K values for kNNs.

The random forest algorithm has been tested with different numbers of trees.

The tuning of parameters for all these three methods were determined *via* grid search optimization on each of the training sets, as implemented in the MATLAB toolboxes.

## Results

### Classification Accuracy

Since there was a problem of acquisition with cardiac or ocular sensors for 5 participants, only the data of 17 participants were retained to achieve the supervised learning of physiological states. After raw data was cleansing and normalization, mean values of pupillary diameter and HRV were calculated every second. The supervised learning was carried out with 2 or 3 categories, at two different layers, one on all participants and one on each participant independently. For the first classification (all participants), the learning was achieved from the data of 13 participants, and the data of the 4 left participants were used as a test sample to check the accuracy of the process. For the second one (for each participant), 75% of the data was used as training sample, and the resulting classification was then tested on the remaining 25% of the data.

The three different chosen methods (SVM, kNN, and RF) were tested with different parameters.

As mentioned in Table 3, SVM and RF produced better classification over all participant data than kNN method, for both 3-classes (respectively 61, 58, and 49%) and binary classification (respectively 83, 79, and 77%).

**TABLE 3:** Accuracy of classification over all participants.

	Supervised learning algorithms	Best setting	Global accuracy	Accuracy of low class	Accuracy of medium class	Accuracy of high class
**3-class classification**	SVM	Kernel sigmoid (gamma = 0.5 and coeff = 0)	61%	55%	13%	100%
	kNN	Chebychev distance and *k* = 1	49%	76%	47%	46%
	RF	68 trees	58%	89%	49%	74%
**2-class classification**	SVM	Kernel sigmoid (gamma = 0.5 and coeff = 1)	83%	54%		95%
	kNN	Chebychev distance and *k* = 18	77%	49%		91%
	RF	23 trees	79%	78%		88%

**TABLE 4:** Accuracy of classification performed individually on each of the 17 participants.

	Supervised learning algorithms	Best settings	Average global accuracy	Standard deviation	Minimum global accuracy	Maximum global accuracy
**3-classes classification**	SVM	Kernel sigmoid (gamma = 0.5 and coeff = 0)	78%	12%	58%	100%
	kNN	Chebychev distance and *k* = 1	74%	14%	55%	100%
	RF	68 arbres	44%	8%	33%	63%
**2-classes classification**	SVM	Kernel sigmoid (gamma = 0.5 and coefficient = 1)	91%	9%	63%	100%
	kNN	Chebychev distance and *k* = 18	89%	9%	63%	100%
	RF	23 trees	69 %	12%	42%	95%

**FIGURE 3:**
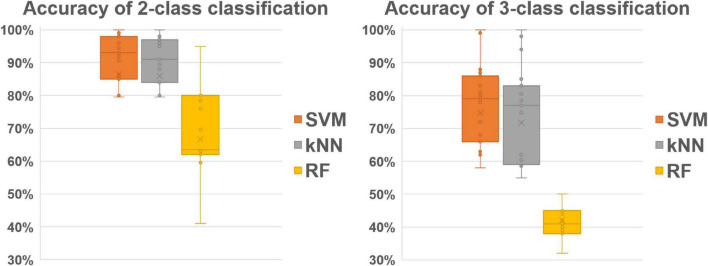
Bar and whisker plot for individual 2-class **(left)** and 3-class **(right)** OFS classification.

For the classification of individual data, Table 4 shows that SVM and kNN are more accurate than RF method for producing 3-classes (respectively 78, 74, and 44% of mean accuracy) and binary classification (respectively 91, 89, and 69% of mean accuracy). Standard deviations of accuracy are also reported in Table 4 and Figure 3 represents the interquartile ranges and the medians of accuracy for the 2-class and 3-class classification over the 17 participants.

Among all the tested methods, the SVM thus give the best results. In addition, the binary classification by the SVM also give better results than the classification in three classes (83 versus 61% on all participants, and 91 versus 78% when data are processed individually).

### Validation of the Operator Functional State Classification

The classification was therefore *a posteriori* validated, by comparing the resulting OFS values with the subjective values collected on ISA questionnaire, collected every 90 s during the scenario. The contingency table (cf. Table 5) between OFS classes and ISA level points out that an OFS with a low risk level correspond to more than 50% of ISA level 1, an OFS with medium risk level matches with more than 53% of ISA levels 2 and 3, and an OFS with high risk level corresponds to more than 68% of ISA levels 4 and 5. Moreover, we analyzed whether the OFS rank was associated with ranking on the ISA questionnaire on these temporal points. Based on the results of the study, those with higher OFS ranks were more likely to have scores that ranked higher on the ISA questionnaire, *r*_τ_ = 0.42, *p* < 0.05.

**TABLE 5:** Operator Functional State contingency table for OFS and ISA.

		OFS		
		OFS 1 = low risk	OFS 2 = medium risk	OFS 3 = high risk
ISA	ISA 1	52%	16%	1%
	ISA 2	33%	30%	8%
	ISA 3	11%	23%	23%
	ISA 4	4%	22%	44%
	ISA 5	0%	10%	24%
Total		27	195	104

## Discussion

This article aims at operationalizing the OFS concept developed by Hockey (2003) and the related approach proposed by Yang and Zhang (2013). Several contributions, limitations and perspectives can be listed on methodological and practical sides.

The present study applied different supervised learning techniques (SVM, RF, and kNN), to classify OFS according to three different categories. Moreover, compared to many studies that arbitrarily and *a priori* set the level of task difficulty, the proposed classification method uses dynamic and objective task difficulty labels to supervise data learning, and these labels were cross validated by the subjective experience expressed by participants.

This present study also showed that OFS estimation in three classes (low, medium, or high risk of control loss) can be significantly associated with the subjective experience of participants assessed with ISA technique. Moreover, the results of supervised learning methods on VHR and DP signals to classify physiological states related to OFS showed that the SVM method, and to a lesser extent the kNN method, produced robust classifications. The Random Forest method appears to be much less efficient. It should also be noted that these results are very similar to those found in the literature.

Table 6 thus positions the results of the present study in relation to the other works of the literature. This implementation therefore shows that the envisaged classification of the cognitive and functional states of an operator carrying out a supervising activity on a adjustable autonomous system is possible.

**TABLE 6:** Comparison between the present study and literature works for 2-class and 3-class OFS classification.

Authors	Data	Number of classes	Supervised learning method	Accuracy
Gagnon et al. (2016)	HR, BR, Gaze position, PD, BLKf, HRV	2	kNN	Individually: 91% All subjects: 89%
			SVM	Individually: 84% All subjects: 83%
Gilani (2016)	HRV, EEG	2	kNN	All subjects: 81%
			SVM	All subjects: 56%
			RF	All subjects: 87%
Yin and Zhang (2014)	EEG, ECG, and EOG	2	LS-SVM	Individual: 93%
		3	SVM (1 vs. 1)	Individual: 72%
Zhang et al. (2015)	EEG, ECG, and EOG	3	SVM	Individual: 69%
**Current study**	HRV, PD	**2**	**SVM**	**Individually: 92% All subjects: 83%**
			**kNN**	**Individually: 90% All subjects: 77%**
			**RF**	**Individually: 70% All subjects: 80%**
		**3**	**SVM**	**Individually: 78% All subjects: 61%**
			**kNN**	**Individually: 75% All subjects: 50%**
			**RF**	**Individually: 40% All subjects: 58%**

*Bold values refer to the results of our own study.*

If the learning methods get a better performance for the binary classification, we can notice that the 3-categories classification remains above 78% (at the individual layer) and 61% (for all participants) in the case of the SVM method and seems better than those obtained in the works of Ying and Zhang (Hancock et al., 1995) and Zhang et al. (2015) with the same method, which did not exceed 72% at the individual layer. Moreover, the accuracy indicated above could be improved by refining the SVM settings or by considering a stacking method that mobilizes several aggregated classification methods. Stacking method would certainly allow to benefit from the contrasting performance of the three selected methods: SVM is very good for the strong class, where the kNN and RF methods seem much better for the low and medium classes, see Table 6).

Finally, the obtained results of this classification approach based on the combination of objective indicators could be implemented for monitoring online operator states and dynamically providing assistances to operators.

Thus, the obtained OFS classification could be considered to trigger an assistance, and the type of countermeasures to be provided will be adapted according to the detection of a specific OFS level (for instance, medium risk, and high risk OFS would not call the same solutions). That would be very helpful for the drone swarm monitoring activity studied in this article, and that could be implemented to other domains, with civilian and military applications for operators involved in risky missions (e.g., nuclear plant or air traffic management). However, an additional challenge to reach such an adaptable human-machine cooperation will be to propose the least intrusive sensors, so as not to interfere with the activity, but also to make acceptable the use of biofeedback sensors as well as the concept that an operator is observed by the system.

## Author’s Note

AK is Doctor in Automatics from Université Bretagne Sud. PR is Associate Professor at Université Bretagne Sud, his research deals with human-autonomy teaming in dynamic situations, and operator cognitive state monitoring. GC is Professor at IMT Atlantique, he mainly works human-machine dialogue to interact with complex and autonomous systems.

## Data Availability Statement

The raw data supporting the conclusions of this article will be made available by the authors, without undue reservation.

## Ethics Statement

Ethical review and approval was not required for the study on human participants in accordance with the local legislation and institutional requirements. The patients/participants provided their written informed consent to participate in this study.

## Author Contributions

AK conducted the studies and collected and analyzed the data. GC and PR supervised the project and carried out the literature review on mental state classification and machine learning methods. All authors wrote the manuscript, under the coordination of PR.

## Conflict of Interest

The authors declare that the research was conducted in the absence of any commercial or financial relationships that could be construed as a potential conflict of interest.

## Publisher’s Note

All claims expressed in this article are solely those of the authors and do not necessarily represent those of their affiliated organizations, or those of the publisher, the editors and the reviewers. Any product that may be evaluated in this article, or claim that may be made by its manufacturer, is not guaranteed or endorsed by the publisher.
